# Fibromyalgia comorbidity in Systemic Lupus Erythematosus patients: assessing impact on quality of life

**DOI:** 10.1186/s42358-024-00432-5

**Published:** 2024-12-18

**Authors:** Jade A. M. Monteiro, Alexia L. H. Gama, Joao C. S. Oliveira, Matheus V. Falcao, Ana Karla G. Melo, Danielle C. S. Egypto, Alessandra S. Braz

**Affiliations:** Department of Internal Medicine, Federal University of Paraiba, João Pessoa, Paraiba 58050-085 Brazil

**Keywords:** Fibromyalgia, Systemic Lupus Erythematosus, Health-related quality of life

## Abstract

**Introduction:**

The prevalence of Fibromyalgia in patients with Systemic Lupus Erythematosus (SLE) is significantly higher compared to the general population. Despite this frequent association, Fibromyalgia remains underdiagnosed and consequently inadequately treated, negatively affecting the quality of life of these patients.

**Objective:**

This study aims to evaluate the occurrence of Fibromyalgia and its impact on the quality of life of Brazilian patients with SLE treated at a University Hospital in the state of Paraiba.

**Materials and methods:**

This descriptive, observational, and cross-sectional study included patients with SLE diagnosed according to the 2012 criteria of the Systemic Lupus Erythematosus International Collaborating Clinics (SLICC). The occurrence of Fibromyalgia was assessed using the American College of Rheumatology (ACR) criteria of 1990 and 2010/2011, revised in 2016. Quality of life was evaluated using the Short-Form 36 (SF-36) questionnaire for all patients, while the Fibromyalgia Impact Questionnaire (FIQ) was applied to those diagnosed with Fibromyalgia.

**Results:**

The sample comprised 107 SLE patients, with an average age of 54.1 years (SD:12.1), of whom 95.4% (102) were women. The prevalence of Fibromyalgia among SLE patients was 19.1% (21), all of whom were women with a mean age of 45.6 years (SD 9.6). The SF-36 scores of SLE patients with Fibromyalgia were consistently lower across all eight domains compared to those without Fibromyalgia, indicating a significant negative impact of this comorbidity. Conclusion: These findings are consistent with existing literature, highlighting the significant negative impact of Fibromyalgia on the quality of life of patients with SLE.

**Conclusion:**

These findings are consistent with existing literature, highlighting the significant negative impact of Fibromyalgia on the quality of life of patients with SLE.

## Introduction

Fibromyalgia, also known as Generalized Pain Amplification Syndrome, is a condition affecting the central processing of pain signals, leading to widespread and persistent musculoskeletal pain, along with symptoms such as allodynia and hyperalgesia. Although pain is its main characteristic, Fibromyalgia has a complex and diverse symptomatology, involving fatigue, headache, sleep disorders, mood changes, weakness and paresthesias [1].

In Brazil, Fibromyalgia affects approximately 2.5% of the population, making it the second most prevalent rheumatological disorder after Osteoarthritis [2]. Globally, its prevalence hovers around 2.7%, with a marked predilection for females, who are afflicted at a ratio of 3:1 compared to males [3]. Moreover, Fibromyalgia frequently co-occurs with other conditions, including both rheumatological disorders like Rheumatoid Arthritis and non-rheumatological ones like depression and irritable bowel syndrome [4].

Among rheumatological conditions, Fibromyalgia often accompanies diseases such as Rheumatoid Arthritis, Ankylosing Spondylitis, Sjogren’s Syndrome, and Systemic Lupus Erythematosus (SLE) [5–9]. Recent research suggests that Fibromyalgia affects 8 to 14% of patients with rheumatological conditions in general [10], with its prevalence among those with SLE even higher, reaching approximately 25% [9, 11, 12]. In Brazil, specifically, a study reported a 12% occurrence of Fibromyalgia among lupus patients [13].

SLE is a chronic inflammatory disease of an autoimmune nature, characterized by the production of autoantibodies and tissue deposition of immune complexes, with activation of cytokines and the complement [14]. It mainly affects women of reproductive age, reaching a ratio of 9 women to 1 man. The prevalence of SLE in the Brazilian population is 98 cases per 100,000 inhabitants [2]. The incidence of the disease in Brazil, in turn, was evaluated in a study carried out in the northeast region, with a total of 8.7 new cases per 100,000 inhabitants/year [15]. This value was at least twice as high as those found in similar foreign studies [16, 17]. Ethnic and environmental factors, such as greater exposure to sunlight, appear to contribute to these observed differences.

The presence of Fibromyalgia is a confounding factor for both diagnosis and assessment of lupus activity, due to the presence of clinical and epidemiological characteristics in common between the two diseases [18]. Early and accurate diagnosis of Fibromyalgia is crucial for guiding appropriate therapeutic interventions, as it significantly impacts patients’ quality of life. Studies indicate that individuals with both Fibromyalgia and SLE experience greater physical and psychosocial impairments com- pared to those with SLE alone [13]. Thus, addressing Fibromyalgia alongside SLE treatment is essential for improving overall quality of life, particularly for patients experiencing persistent symptoms despite SLE control [9, 13, 18].

Given the profound social implications of chronic pain syndromes, our study aimed to assess the prevalence of Fibromyalgia among SLE patients treated at a University Hospital in Paraiba, Brazil, and to evaluate its impact on their quality of life.

## Methods

This study employed a descriptive, observational, cross-sectional design and was conducted at the rheumatology outpatient clinic of Lauro Wanderley University Hospital (HULW) in João Pessoa, Paraiba, Brazil. Sampling involved non-probabilistic techniques to recruit patients diagnosed with SLE between August 2017 and June 2018. Sample size estimation relied on the prevalence of SLE in the general population and the reported frequency of Fibromyalgia comorbidity in Brazilian studies [2, 13]. Inclusion criteria encompassed patients meeting the SLICC-2012 criteria for SLE diagnosis [19]. Exclusion criteria comprised patients with cognitive deficits, severe psychiatric disorders, neurological sequelae, or existing diagnoses of other autoimmune diseases.

Data collection involved active screening for the ACR 1990 and 2010 Fibromyalgia diagnostic criteria, revised in 2016, among SLE patients [20–22]. Rheumatologists performed tender point palpation. Additionally, all participants completed the Short-Form 36 (SF-36) Quality of Life Assessment questionnaire [23]. Patients meeting Fibromyalgia criteria underwent further assessment using the validated Brazilian version of the Fibromyalgia Impact Questionnaire (FIQ) [24].

The study protocol was approved by the Research Ethics Committee of the Federal University of Paraiba (approval number 036996/2017). Informed consent was obtained from all participants prior to any data collection procedures. The study adhered to relevant guidelines and regulations throughout its duration, ensuring the ethical conduct of research involving human participants. Participants were briefed on the study objectives, procedures, potential risks, and benefits before providing written consent. Statistical analyses employed descriptive statistics to summarize sociodemographic and clinical characteristics, including frequencies for categorical variables and means with standard deviations (SD) for parametric scalar variables or medians with interquartile ranges (IQR) for non-parametric variables. The Shapiro-Wilk test assessed normality. Inferential statistics included Student’s *t*-test or Mann-Whitney test for quantitative variables. The significance level was set at 0.05.

## Results

The sample comprised 107 patients diagnosed with SLE according to the SLICC 2012 criteria. The average age of all patients in the study is 43.4 years, with 95.4% (102) being women. Both the 1990 and the 2016 revisions of the 2010/2011 ACR criteria for Fibromyalgia were applied to all patients in the sample.

Following the application, 21 patients were classified as having Fibromyalgia, representing a frequency of 19.1%, with a 95% confidence interval (10.69–28.57%). Currently, after the updates of 2016, Fibromyalgia diagnosis entails Widespread pain index (WPI) ≥ 7 + Symptom Severity Scale (SSS) ≥ 5 or WPI 4–6 + SSS ≥ 9, with symptoms at a similar level for at least 3 months [22]. All 21 Fibromyalgia-diagnosed patients in our study met this criterion. 17 patients had at least 11 of the 18 tender points, meeting the 1990 criteria, while 4 received diagnoses solely based on the newer criteria, which do not require the presence of tender points.

Subsequently, the sample was segregated into two groups: patients with SLE alone (Group 1) and those with SLE and Fibromyalgia (Group 2). Sociodemographic characteristics of both the SLE-only group and the SLE with Fibromyalgia group are detailed in Table 1. All Fibromyalgia-diagnosed patients were women, with a mean age of 45.6 years (SD 9.6). The mean number of tender points was 13.2 (SD 3.1), while the widespread pain index (WPI) averaged 12.1 (SD 1.6). The symptom severity scale (ESS) yielded a mean value of 9.2 (SD 1.5). Among somatic symptoms, 52.4% (11) reported the presence of three symptoms (headaches, pain or cramps in the lower abdomen, and depression), while 47.6% (10) reported one to two symptoms. No patients reported having no symptoms. Table 2 presents the diagnostic criteria from the SLICC 2012 guidelines for both groups.

**Table 1:** Sociodemographic characteristics of SLE patients with and without Fibromyalgia

Only SLE	SLE and Fibromyalgia
**Age – ** ***avg. ± std. dev.***	
42.9 years ± 12.52	45.6 years ± 9.76
**Gender – ** ***n (%)***	
Female: 80 (94.1%)	Female: 21 (100%)
Male: 5 (5.9%)	
**Ethnicity – ** ***n (%)***	
White:12 (14.1%)	White: 2 (9.5%)
Black: 10 (11.8%)	Black: 4 (19.1%)
Brown: 63 (74.1%)	Brown: 15 (71.4%)
**Marital status – ** ***n (%)***	
Married: 41 (48.2%)	Married: 13 (61.9%)
Single: 33 (38.8%)	Single: 5 (23.8%)
Divorced: 6 (7.1%)	Divorced: 2 (9.5%)
Widowed: 5 (5.9%)	Widowed: 1 (4.8%)
**Education – ** ***n (%)***	
Elementary: 39 (45.9%)	Elementary: 11 (52.4%)
Secondary: 39 (45.9%)	Secondary: 7 (33.3%)
Post-Secondary: 7 (8.2%)	Post-Second.: 3 (14.3%)
**Working – ** ***n (%)***	
Yes: 29 (34.1%)	Yes: 4 (19.1%)
No: 56 (65.9%)	No: 17 (80.9%)

**Table 2:** Comparison of SLICC-2012 diagnostic criteria between lupus only and lupus with Fibromyalgia patients

Criteria	Only SLE	SLE + Fibromyalgia
Acute cutaneous lupus	65 (75.6%)	17 (81%)
Chronic cutaneous lupus	7 (8.1%)	2 (9.5%)
Alopecia	52 (60.5%)	15 (71.4%)
Oral ulcers	23 (26.7%)	5 (23.8%)
Arthritis	74 (86%)	18 (85.7%)
Serositis	20 (23.3%)	2 (9.5%)
Nephritis	17 (19.8%)	4 (19%)
Neurological	6 (7%)	1 (4.8%)
Positive ANA	82 (95.3%)	20 (95.2%)
Anti-DNA	15 (17.4%)	3 (14.3%)
Anti-SM	10 (11.6%)	4 (19%)
Low Complement	8 (9.3%)	1 (4.8%)

**Fig. 1:**
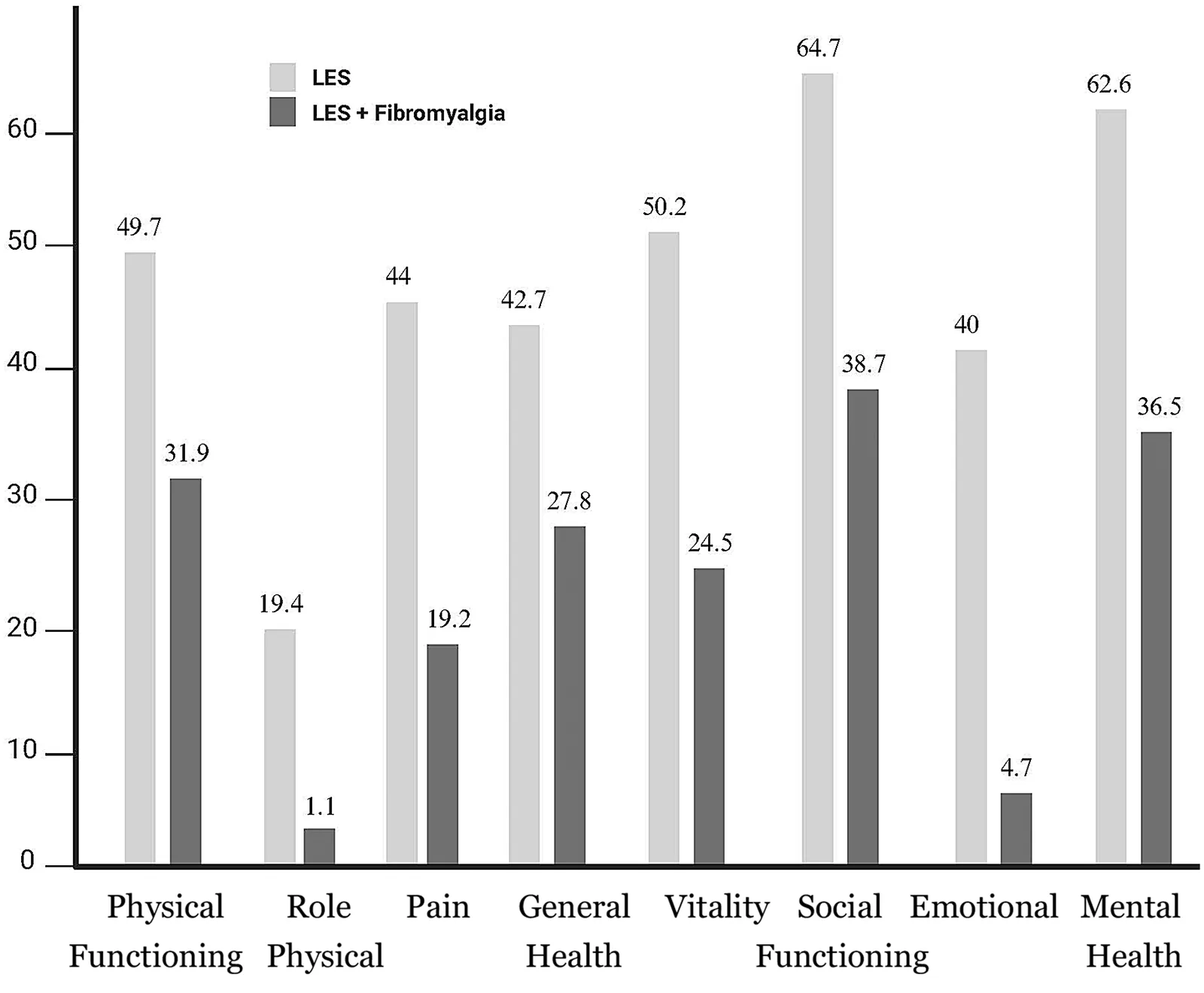
Average scores obtained in the domains of the SF-36 questionnaire among patients with and without Fibromyalgia

Evaluation of the impact of Fibromyalgia using the FIQ revealed an average score of 66.2 (SD 12) on a scale of 0 to 100. Among the 10 variables assessed by the questionnaire, pain and fatigue emerged as the most significant concerns among patients in the sample. Both groups underwent assessment using the SF-36 quality of life questionnaire, with subsequent comparison of results. The group with SLE and Fibromyalgia exhibited poorer scores across all eight domains of the questionnaire when compared to the SLE-only group. Notably, physical and emotional aspects were particularly affected by Fibromyalgia. Figure 1 illustrates the average scores obtained by both groups in each SF-36 domain. The adverse impact of Fibromyalgia was statistically significant across all of them.

## Discussion

Fibromyalgia symptomatology extends beyond generalized pain to encompass a spectrum of heterogeneous and nonspecific manifestations. Given the subjective nature and the absence of biomarkers, diagnoses poses a challenge. In the context of SLE, diagnostic is even more complex. The presence of Fibromyalgia complicates both the diagnosis and evaluation of SLE activity, given the overlapping clinical manifestations shared by both conditions. In this study, we investigated the prevalence of Fibromyalgia among Brazilian patients with Systemic Lupus Erythematosus (SLE) and its impact on their quality of life. Employing both the 1990 and the revised 2010/2011 ACR criteria enhances diagnostic accuracy and ensures a comprehensive assessment of Fibromyalgia [25]. This research addresses a significant gap in understanding comorbidities in this population, highlighting the substantial burden that Fibromyalgia imposes on the quality of life of SLE patients.

Fibromyalgia was identified in 19.1% (21) of the SLE patients included in our study. This prevalence aligns with observations from previous studies, which reported Fibromyalgia frequencies among SLE patients hovering around 22% [9, 11, 12]. Contrasting with prior Brazilian data which reported a 12% prevalence of Fibromyalgia in SLE patients, this current investigation unveiled a notably higher prevalence. The earlier Brazilian study examined a smaller patient sample, segregating them into three categories: Fibromyalgia, SLE, and Fibromyalgia + SLE. Their findings indicated that while Fibromyalgia did not exacerbate SLE activity rates, it notably impaired the quality of life in SLE patients [13]. No other comparable studies assessing the Brazilian population are documented in the literature.

When comparing the sociodemographic characteristics of SLE patients with and without Fibromyalgia in our sample, notable differences emerge. The average age of SLE patients without Fibromyalgia is 42.9 years (± 12.52), while those with Fibromyalgia are slightly older, averaging 45.6 years (± 9.76). In terms of gender, the group without Fibromyalgia consists of 94.1% females and 5.9% males, while the group with Fibromyalgia is entirely female. Education levels are similar between the groups; however, a higher percentage of patients without fibromyalgia are employed (34.1%), compared to only 19.1% in the fibromyalgia group. These differences underscore the negative social impact of fibromyalgia on patients with lupus, particularly in terms of employment and daily functioning.

In comparing the two groups based on the SLICC-2012 criteria for lupus, most clinical criteria were found to be similar. However, the group with fibromyalgia exhibited a higher prevalence of acute cutaneous manifestations, such as malar rash, with 81% (17 patients) compared to 75.6% (65 patients) in the lupus-only group. Additionally, alopecia was more common in Group 2, affecting 71.4% (15 patients) versus 60.5% (52 patients) in Group 1. Both groups showed similar rates of chronic cutaneous involvement, arthritis, renal issues, serositis, and neurological manifestations. Immunological profiles were also comparable. In Group 1, 95.3% (82 patients) had positive antinuclear antibodies (ANA), with 17.4% (15 patients) showing anti-DNA antibodies and 11.6% (10 patients) positive for Anti-SM. Group 2 had 95.2% (20 patients) testing positive for ANA, 14.3% (3 patients) for anti-DNA and 19% (4 patients) for anti-SM.

A very recent study [26] yielded results consistent with our findings. The authors compared the clinical phenotype-laboratory characteristics between lupus patients with and without Fibromyalgia. No differences were noted between the groups, except for a higher incidence of malar rash and active arthritis in Fibromyalgia patients, despite similar autoantibody profiles. The study also found no correlation between fibromyalgia and disease activity, as assessed by the Systemic Lupus Erythematosus Disease Activity Index 2000 (SLEDAI-2k) [27]. This finding aligns with numerous previous studies [5, 11, 13, 18, 28, 29].

Fibromyalgia diagnosis relies on criteria outlined by the American College of Rheumatology (ACR). The 1990 criteria primarily require diffuse pain presence (above and below the waist, right and left sides, and axial) and tender point examination, with at least 11 of the 18 sites being tender [20]. In our study, 15.8% (17) of patients exhibited 11 or more tender points, slightly lower than another study’s findings, which concluded that 17.3% of SLE patients had 11 or more tender points. The American study further revealed worsened health status, as assessed using the Health Assessment Questionnaire, in SLE patients with a higher number of tender points, even without Fibromyalgia diagnoses [30].

Given Fibromyalgia’s multifactorial nature and symptomatic heterogeneity, questionnaires are indispensable for objectively assessing affected patients’ quality of life. The Fibromyalgia Impact Questionnaire (FIQ) is widely employed for this purpose due to its simplicity and effectiveness. This tool gauges functional capacity, employment status, psychological disorders, and physical symptoms, proving reliable in evaluating Fibromyalgia therapeutic efficacy [31]. First published in 1991 and subsequently revised in 1997 and 2002, the FIQ was culturally adapted for the Brazilian population and validated in 2006 [24]. Among Fibromyalgia patients assessed with the FIQ in our study, pain and fatigue emerged as the most detrimental to quality of life, corroborating findings from a prior Brazilian study [13].

The SF-36 questionnaire assesses quality of life across eight domains: functional capacity, physical aspects, pain, general health status, vitality, social aspects, emotional aspects, and mental health. Application of the SF-36 in the present study revealed lower scores across all eight domains among SLE patients with Fibromyalgia compared to those without. This pronounced negative impact of Fibromyalgia on lupus patients’ quality of life echoes findings from previous studies using the same instrument [29, 32]. These data underscore the criticality of early Fibromyalgia diagnosis and appropriate therapeutic management to enhance SLE patient well-being.

Studies evaluating Fibromyalgia’s impact on SLE patients’ quality of life using alternative tools have also demonstrated the syndrome’s adverse effects. A 2009 Spanish study uncovered an association between Fibromyalgia and anxious and depressive psychiatric symptoms. Utilizing the Short Form 12 questionnaire (SF-12), the study reported lower scores in both the physical and mental components among Fibromyalgia- afflicted individuals [33, 34]. Valencia-Flores et al. [35] evaluated Fibromyalgia and SLE patients’ functional statuses using the Health Assessment Questionnaire Disability Index (HAQ-DI), validated for their demographic. Fibromyalgia patients exhibited higher disability rates, alongside increased occurrences of sleep disturbances, fatigue, and depression.

The 2019 European Alliance of Associations for Rheumatology (EULAR) recommendations for SLE management include enhancing Health-Related Quality of Life (HRQoL) as a treatment objective [36]. While remission or low disease activity are ideal therapeutic goals, conflicting data regarding their impact on HRQoL improvement exist in the literature. A recent study highlights Fibromyalgia as a contributor to discordance between SLE patients’ self-perceived health statuses and physician-reported disease activity measures [37]. These findings emphasize the necessity of early Fibromyalgia diagnosis and proper comorbidity management to foster SLE patient well-being.

The study faced several limitations. Despite employing questionnaires validated for the Brazilian population, several patients experienced challenges in understanding the questionnaires, which may have affected their responses. Additionally, we did not evaluate disease activity in SLE using the SLEDAI-2k as a variable in our study, nor did we assess the SLICC/ACR Damage Index (SDI). Another significant limitation was the absence of a healthy control group and a group consisting solely of patients with Fibromyalgia.

## Conclusions

The findings of our study align with existing literature, showing Fibromyalgia as a prevalent and underdiagnosed condition among individuals with SLE, exerting a detrimental impact on their quality of life. Given the significant physical, psychological, and socioeconomic ramifications of chronic pain syndromes, accurate identification of Fibromyalgia stands as a crucial step in the comprehensive management of pain, fatigue, and somatic symptoms frequently encountered in SLE patients.

Understanding the high prevalence of Fibromyalgia within the SLE population, alongside the reliance on exclusively clinical criteria for diagnosis, is crucial for discerning between symptoms originating from Fibromyalgia and those reflective of SLE activity. This differentiation is essential for directing suitable therapeutic interventions to alleviate symptoms while minimizing potential iatrogenic consequences, ultimately contributing to the improvement of overall quality of life for affected individuals.

## Data Availability

Data is provided within the manuscript.
